# Integrative analysis of single-cell and bulk transcriptome data reveal the significant role of macrophages in lupus nephritis

**DOI:** 10.1186/s13075-024-03311-y

**Published:** 2024-04-12

**Authors:** Shuping Wei, Haiyun Shen, Yidan Zhang, Chunrui Liu, Shoushan Li, Jing Yao, Zhibin Jin, Hongliang Yu

**Affiliations:** 1 Department of Ultrasound, Nanjing Drum Tower Hospital, Affiliated Hospital of Medical School, Nanjing University, 321 Zhongshan Road, Nanjing, 210008 Jiangsu PR China; 2 Department of oncology, The Siyang Hospital of Chinese Traditional Medicine, 15 Jiefangbei Road, Zhongxing district, Siyang country, Suqian, 223798 Jiangsu PR China; 3 Department of radiation oncology, The Affiliated Cancer Hospital of Nanjing Medical University & Jiangsu Cancer Hospital & Jiangsu Institute of Cancer Research, 42 Baiziting Road, Nanjing, 210007 Jiangsu PR China

**Keywords:** Lupus nephritis, ScRNA-seq, Infiltrating immune cells, Macrophages, LGALS9

## Abstract

**Objective:**

We attempted to identify abnormal immune cell components and signaling pathways in lupus nephritis (LN) and to identify potential therapeutic targets.

**Methods:**

Differentially expressed genes (DEGs) between LN and normal kidney tissues were identified from bulk transcriptome data, and functional annotation was performed. The phenotypic changes in macrophages and aberrant intercellular signaling communications within immune cells were imputed from LN scRNA-seq data using trajectory analysis and verified using immunofluorescence staining. Finally, lentivirus-mediated overexpression of LGALS9, the gene encoding Galectin 9, in THP-1 cells was used to study the functional effect of this gene on monocytic cells.

**Results:**

From bulk transcriptome data, a significant activation of interferon (IFN) signaling was observed, and its intensity showed a significantly positive correlation with the abundance of infiltrating macrophages in LN. Analysis of scRNA-seq data revealed 17 immune cell clusters, with macrophages showing the highest enrichment of intercellular signal communication in LN. Trajectory analysis revealed macrophages in LN undergo a phenotypic change from inflammatory patrolling macrophages to phagocytic and then to antigen-presenting macrophages, and secrete various pro-inflammatory factors and complement components. LGALS9 was found significantly upregulated in macrophages in LN, which was confirmed by the immunofluorescence assay. Gene functional study showed that LGALS9 overexpression in THP-1 cells significantly elicited pro-inflammatory activation, releasing multiple immune cell chemoattractants.

**Conclusion:**

Our results present an important pathophysiological role for macrophages in LN, and our preliminary results demonstrate significant pro-inflammatory effects of LGALS9 gene in LN macrophages.

**Supplementary Information:**

The online version contains supplementary material available at 10.1186/s13075-024-03311-y.

## Key message

1. Macrophages were found massively infiltrate in the parenchyma of LN kidney.
2. Macrophages play a vital role in aberrant intercellular signaling in LN and undergo a significant phenotypic evolution in LN.
3. This study suggests for the first time that LGALS9 could be a potential druggable target in macrophage targeting strategy in treating LN.

**Supplementary Information:**

The online version contains supplementary material available at 10.1186/s13075-024-03311-y.

## Introduction

Lupus nephritis (LN) is a lethal complication of the autoimmune disease, systemic lupus erythematosus. Glucocorticosteroids and immunosuppressants are the cornerstones of treatment for this disease; however, long-term treatment can be toxic and ineffective. The emerging novel monoclonal antibody drugs, anifrolumab and belimumab, are still ineffective, as they can only elicit a response rate benefit of approximately 15% compared to that with the placebo at 52 weeks, and more than 50% of the patients do not respond to these agents [1, 2]. More comprehensive investigations of the molecular pathways that drive LN could lead to new, and effective treatments for LN.

Macrophages are highly plastic immune cells that can alter their functional state in response to microenvironmental signals. Broadly, macrophages are categorized into two types: M1 (classically activated) and M2 (alternatively activated), although this classification is an oversimplification, as macrophages exhibit a spectrum of activation states. M1 macrophages are typically pro-inflammatory, exacerbating tissue damage in inflammatory conditions, while M2 macrophages participate in tissue repair and immunoregulation [3]. In renal diseases, macrophages can either exacerbate or alleviate disease progression. In acute kidney injury, M1 macrophages may intensify tissue damage through inflammatory actions, whereas M2 macrophages aid in tissue repair [4]. In chronic kidney disease, macrophages contribute to sustained inflammation, fibrosis, and worsening of renal damage [5]. Therefore, therapeutic strategies targeting macrophage activation and plasticity are being explored as potential treatments for various kidney diseases.

In this study, we revealed an important pathophysiological role of macrophages in LN by integrative analysis of both single-cell and bulk transcriptome data of LN. Our results showed the abundance of macrophages was positively correlated with the intensity of the IFN signaling pathway in LN. They play an important role in aberrant autoantigen presentation in LN and secrete multiple pro-inflammatory factors. Additionally, cell-cell communication analysis showed macrophages in LN had significant enrichment of LGALS9 pathway. Gain of function study showed overexpression of LGALS9, the gene encoding Galectin 9, in monocytic THP-1 cells had significant pro-inflammatory phenotypic effects, such as promotion of cell migration, phagocytosis, and secretion of multiple inflammatory mediators, suggesting its vital role in the aberrant activation of macrophages in LN. Therefore, our study systematically presents an important pathophysiological role of macrophages in LN and demonstrate significant pro-inflammatory effects of LGALS9 gene in LN macrophages.

## Methods

### Statistical analysis

All analyses were conducted using R (version 4.1.1) and SPSS (version 25.0). All comparisons for continuous variables were performed using the two-sided Mann–Whitney test for two groups and the Kruskal–‒Wallis test for more than two groups. The chi-square test or Fisher’s exact test was used for categorical variables. P-values were adjusted using the Benjamini-Hochberg procedure, and the associated false discovery rate (FDR) values were calculated. Statistical significance was set at *p* < 0.05.

### Public datasets collection

The GSE32591 dataset comprising microarray data of glomeruli and tubulointerstitium components from both LN patients and control normal kidney tissues was downloaded from GEO database (https://www.ncbi.nlm.nih.gov/geo). During sample preparation, renal compartments of glomeruli and tubulointerstitium samples were manually microdissected under a stereomicroscope using two dissection needle holders in an ice-cold solution, following a previously established protocol [6, 7]. The scRNA-seq data were downloaded from Human Cell Atlas database (https://explore.data.humancellatlas.org/projects), including single cell transcriptome from lupus nephritis [8], IgA nephropathy [9] and allograft biopsy specimen [10], and from GEO database for Acute kidney injury and chronic kidney disease [11], with the accession number of GSE183279.

### Determination of the immune landscape from the bulk transcriptome

To comprehensively deconvolute the immune infiltration and IFN signature scores from the bulk transcriptome samples, we employed the “IOBR” package (version 0.99.9) [12], a user-friendly tool integrating several methodologies, including “CIBERSORT” and “EPIC”. The absolute mode of “CIBERSORT” was selected to compare the infiltrating immune cells in different samples. To calculate the linear correlation between macrophage abundance and the expression of each gene, macrophage infiltration was imputed using the “EPIC” method [13] in the IOBR package.

### Gene signatures and scores

Gene signatures were defined as follows: Type I IFN signature: MX1, TNFSF10, RSAD2, IFIT1, IFIT3, IFIT2, IRF7, DDX4, MX2, and ISG20 [14]; IFN-γ signature genes: IDO1, CXCL10, CXCL9, HLA-DRA, STAT1, and IFNG [15]. Signature scores were calculated as the single-sample gene-set enrichment analysis (ssGSEA) scores of genes included in each signature for each sample in gene set GSE32591. The calculations were performed using the “calculate_sig_score” function in the R package “IOBR” (version 0.99.9) [12], using the “ssGSEA” method. To calculate the correlation matrix for IFN scores and 22 immune cell infiltration types, the R package “corrplot” (version 0.84) was applied.

### ssGSEA enrichment of each immune cell type from bulk transcriptome data

When obtaining the enrichment scores of each individual immune cell type identified from the scRNA-seq data analysis in the bulk transcriptome, we first obtained the marker genes of each immune population in scRNA-seq data using the “FindAllMarkers” function in the Seurat package. The ssGSEA score of each cell cluster was then calculated as the same method as in the gene signature scores sector, using the “calculate_sig_score” function in IOBR package, with the “ssGSEA” method.

### scRNA-seq data quality control

The scRNA-seq data were processed using the Seurat package (version 4.1.0) in R. In this study, high-quality cells were defined as described in a previous study [8] which required at least 1000 detected genes per cell and an upper threshold of 5000 detected genes per cell to remove potential multiple cells. The percentage of reads mapped to mitochondrial genes per cell was set below 5% in this study. To eliminate batch effects, we applied the Harmony package (version 0.1.0) in R language.

### Transferring cell type labels from reference LN data to PBMC scRNA-seq data

First, we found highly variable features in the two datasets using the “FindVariableFeatures” function in Seurat. We used the top 5000 variable features as the input for integration, resulting in approximately 3000 variable features for downstream analysis. We found anchors between the reference LN data and PBMC scRNA-seq data using the “FindTransferAnchors” function with the parameters dims = 1:30 and reduction = ”cca.” Finally, we transferred cell type labels from the reference LN data to the PBMC dataset using the “TransferData” function with parameter dims = 1:30.

### Functional enrichment analysis

GO and KEGG pathway enrichment analyses for corresponding DEGs were performed and visualized using the R package, clusterProfiler (version 4.0.5), and the p value was adjusted using the Benjamin-Hochberg method.

### Cell‒cell communication analysis

To analyze the communication-related interactions between cell populations and to identify the pathway molecules at single-cell resolution, the R package “CellChat” (version 1.1.4) was applied [16] to the results of the 17 immune cell clusters. Using the “aggregateNet” function in CellChat, the aggregated cell-cell communication networks were calculated. The overall signaling strength of each cell group was visualized using the “netAnalysis_signalingRole_heatmap” function.

### Trajectory analysis of multiple macrophage subtypes

To better understand the genomic changes within the macrophage subtypes of LN, we used the R dyno (version 0.1.2) package [17] to perform trajectory inference on the UMAP representations of the three macrophage subtypes. The pseudotime of the scRNA-seq data was inferred using the “slingshot” method. The gene expression variation heatmap along the dynamic biological pseudotime was visualized by applying the “plot_heatmap” function.

### Patients and sample collection

Samples from five patients with biopsy-confirmed lupus nephritis (LN) and five patients with renal clear cell carcinoma were included in the study, all of whom were from Nanjing Drum Tower Hospital. The demographic information of the included patients was summarized in Supplementary Table 1. LN tissues were obtained as kidney needle puncture samples from patients with LN and normal kidney tissues were obtained from tumor-adjacent normal tissues during clear cell renal cell carcinoma surgery, where the adjacent normal tissues were more than 2 cm from the tumor margin. This study was a retrospective analysis, and thus, the informed consent were waived. The study was approved by the Ethics Committee of Drum Tower Hospital after ethical review (No.2022-259-02).

### HE staining and multiplexed immunofluorescence staining

All LN biopsy samples and normal renal control tissues were processed into paraffin blocks, then, the paraffin embedded samples were sliced into 4-µm sections. After deparaffinization and rehydration, the tissues were subjected to hematoxylin and eosin (HE) as well as multiplexed immunofluorescence staining. The detailed procedure has been described elsewhere [18]. Five immune cell classes were probed: B cells (CD19: GB11061, Servicebio), T cells (CD3, GB13440, Servicebio), CD8 + T cells (CD8, GB12068, Servicebio), macrophages (CD68, GB113150, Servicebio), and CD163 + macrophages (CD163, GB13340, Servicebio). The slides were scanned using an automatic digital slide scanner Pannoramic MIDI (3DHISTECH, Hungary) and analyzed using CaseViewer (3DHISTECH, Hungary).

### LGALS9 overexpression in THP-1 cells and downstream functional analysis

Lentivirus of negative control (CON335) and LV- LGALS9 (30842-14) were constructed by GeneChem Corporation (Shanghai, China). Transfection was performed according to the manufacturer’s protocol. Briefly, THP-1 monocytic cells were incubated in 1640 medium with 10% FBS. The cells were then transduced with the recombinant lentivirus in the presence of polybrene. Stably transfected cell lines expressing GFP were screened using puromycin. LGALS9 expression was subsequently evaluated using real-time PCR. The primers used for human LGALS9 were sense, 5′-TCTCCAGGACGGACTTCAGA-3′ and anti-sense, 5′-CACCAGGAAGCAGAGGTCAA-3′. The primers for the internal reference gene ACTB were sense, 5′-GCGTGACATTAAGGAGAAGC-3′ and anti-sense, 5′-CCACGTCACACTTCATGATGG-3′. Total RNA of cell lines from the control group THP-1_NC and experimental group LGALS9_OE were extracted using a RNeasy Mini Kit (Qiagen). Three replicates were used per treatment group in this study. After cDNA library preparation, the libraries were sequenced on Illumina HiSeq 2500 and the resulting FASTQ files were aligned to the human genome (GRCh38.74). Gene expression profile for the individual samples was thereafter calculated as RPKM values.

### ELISA measurement

Cytokine protein levels of CCL3, CCL4 and CXCL8 were measured in cell culture supernatant of THP-1_NC and LGALS9_OE cells, after the equal number cell passaging and culturing for 48 h, using commercially available ELISA kits (Beyotime, Shanghai, China) according to the manufacturer’s protocol. Absorbance was measured at 450 nm with Synergy HT multi-mode reader (Bio-Tek, Vermont, USA). Three replicates were used per treatment group in this study.

### Western blot

The protein expression pattern of Galectin-9, CD163 and CXCR3 in both THP-1_NC and LGALS9_OE cells was evaluated by western blotting. Briefly, cells were seeded in six-well plates and cultured to 70% confluence. Then, whole cell lysates were extracted and prepared with RIPA buffer containing protease inhibitor cocktail. Western blotting was conducted with 100 µg of the protein extract as described elsewhere [19]. Western blot was performed with anti-β-actin (1:1000, TA09, Zhongshan Goldenbridge Biotechnology, Beijing, China), anti-CD163 (1:500, TA506381, Zhongshan Goldenbridge Biotechnology, Beijing, China), anti-Galectin9 (1:200, ab184331, Abcam, Cambridge, UK) and anti-CXCR3 (1:500, ab288437, Abcam, Cambridge, UK).

## Results

1. Bulk RNA-seq data showed a significant IFN signature in LN, the intensity of which was positively correlated with infiltrating macrophage abundance.

**Fig. 1:**
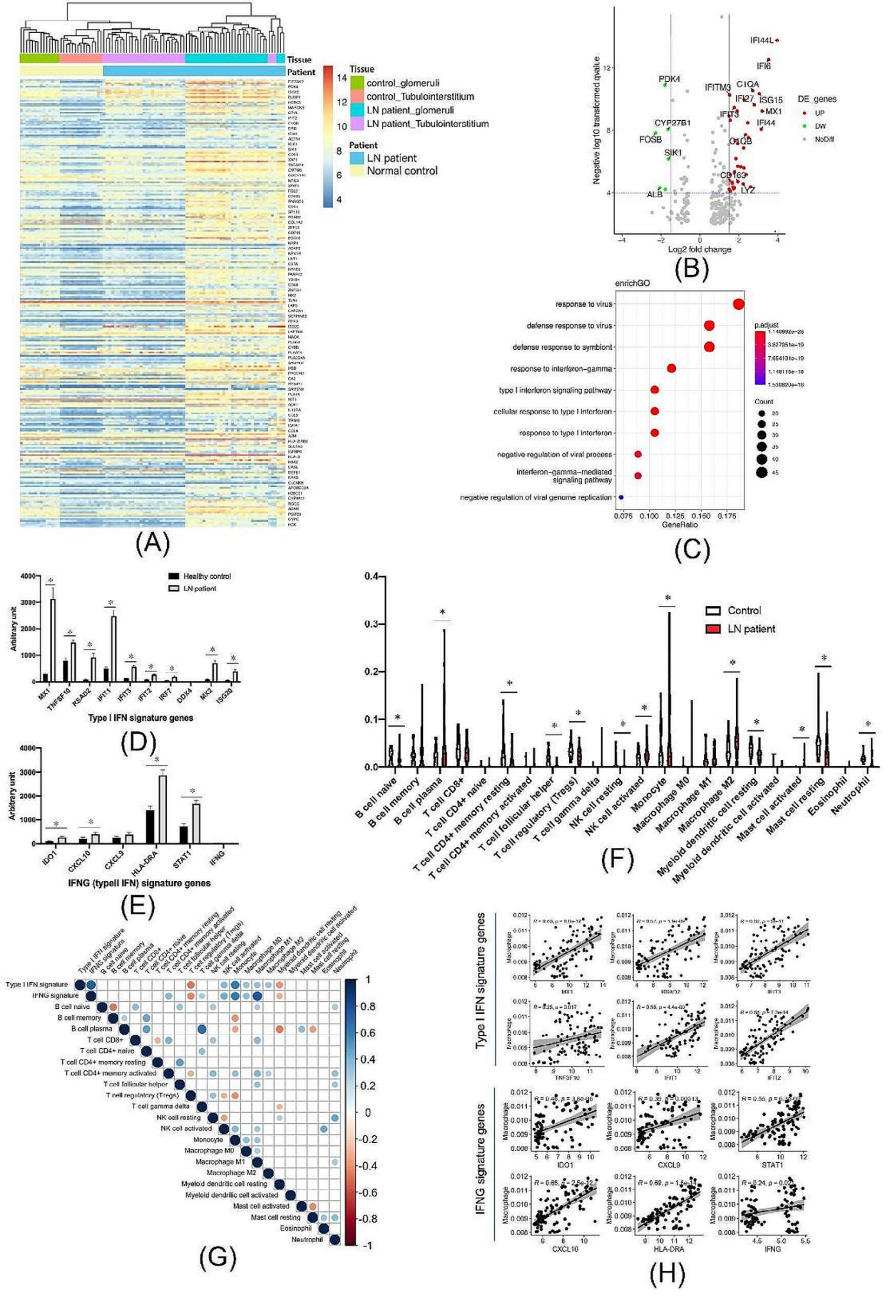
Results of bulk transcriptome analysis for lupus nephritis (LN). (**A**) Heatmap of bulk transcriptome differentially expressed genes (DEGs) between LN and normal kidney tissues. (**B**) Volcano plot of the bulk transcriptome DEGs between LN and normal kidney tissues. (**C**) Bubble plot of the results of gene ontology (GO) enrichment analysis of the DEGs. (**D**, **E**) Interferon (IFN) signature genes were significantly upregulated in LN samples compared with in normal kidney samples. (**F**) The infiltration abundance of up to 22 types of immune cells imputed from bulk transcriptomes of LN and control samples using the CIBERSORT method. (**G**) Correlation matrix of the 22 immune cell types and the IFN scores of LN samples. (**H**) The linear correlations between IFN signature genes and macrophage abundance in LN samples. * p-value less than 0.05

Differential gene expression analysis of bulk transcriptomes between LN and normal kidneys was performed. A heatmap of differentially expressed genes (DEGs) is shown in Fig. 1A. A volcano plot of DEGs is shown in Fig. 1B. Some of the most significantly upregulated genes, including C1QA, C1QB, CD163, and LYZ, were overwhelmingly expressed by infiltrating macrophages, as shown in Supplementary Fig. 1. GO enrichment analysis showed that the DEGs were mainly enriched in response to virus, response to type I IFN, and IFN-γ pathways, as shown in Fig. 1C. A comparison of IFN-stimulated genes (ISGs) [20] between LN and normal kidneys is presented in Fig. 1D and E. Our results indicate that ISG levels were significantly higher in LN than in normal kidney tissues. Using the CIBERSORT algorithm [21], the abundance of up to 22 immune cell types in LN and normal kidneys was imputed from bulk RNA-seq data and is presented in Fig. 1F. A correlation matrix was then calculated between these 22 immune cell types and IFN signature scores, as shown in Fig. 1G. Our results showed that both type I IFN and IFN-γ signature scores were positively correlated with activated NK cells, monocytes, and most macrophages but negatively correlated with regulatory T cells and resting myeloid dendritic cells. The linear relationship between the abundance of total infiltrating macrophages and the expression of Type I IFN and IFN-γ signature genes is shown in Fig. 1H, in which almost all ISGs showed a positive correlation with the abundance of macrophage infiltration in LN.

2. Analysis of scRNA-seq data revealed a vital role for macrophages in aberrant intercellular signaling in LN and significant phenotypic evolution of macrophages in LN.

**Fig. 2:**
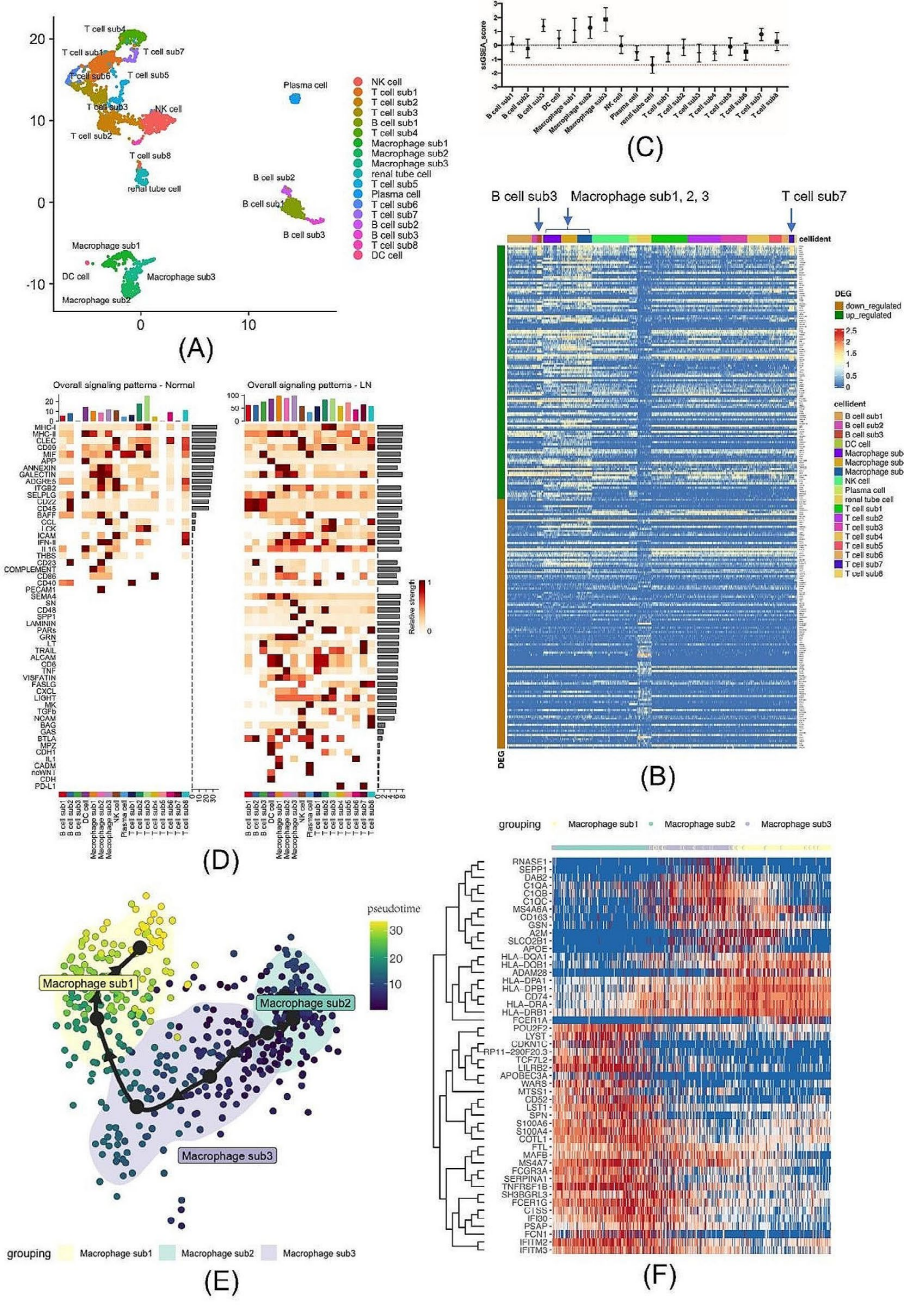
Results of scRNA-seq data analysis of lupus nephritis (LN). (**A**) Uniform manifold approximation and projection (UMAP) plot of the scRNA-seq data of LN with cell type annotations. (**B**) Heatmap of the expression of differentially expressed genes (DEGs) identified from the bulk transcriptome in each cell cluster from scRNA-seq data of LN. (**C**) ssGSEA scores of each cell cluster in LN for the bulk transcriptome DEGs. (**D**) Heatmap of the cellular signaling pathway of each immune cell cluster in LN and normal kidney tissue. (**E**) Results of the trajectory analysis of macrophages in LN, showing the distribution of macrophage subtypes alongside the pseudotime. (**F**) The heatmap of marker genes alongside the evolution of macrophages in LN

For scRNA-seq data analysis, cell filtering and quality control of scRNA-seq data were performed as previously reported by Arazi et al [8]. After clustering with the optimized resolution parameter [22], as shown in Supplementary Fig. 2, a uniform manifold approximation and projection (UMAP) plot of scRNA-seq data recovered from LN tissues labeled by cell type is presented in Fig. 2A. Based on the expression of canonical lineage markers, 18 cell clusters were annotated within the scRNA-seq data, of which 17 and one belonged to leukocytes and kidney epithelial cells, respectively. A heatmap visually presenting the expression of genes of bulk transcriptomic DEGs in each immune cell cluster in scRNA-seq data is presented in Fig. 2B. To quantitatively determine the extent to which each immune cell cluster contributes to the total difference in bulk transcriptomic DEGs, we performed a single-sample gene-set enrichment analysis (ssGSEA) [23, 24]. The normalized enrichment statistics (NES, also called the ssGSEA score) of each cluster were calculated and are presented in Fig. 2C. The results of ssGSEA quantitatively confirmed the gene enrichment pattern observed in the heatmap, that all three subtypes of macrophages had significantly high ssGSEA scores, with macrophage sub3 showing the highest score among all immune cell clusters. Figure 2D is the result of intercellular signal communication pattern analysis in the normal kidney and LN. We found that macrophages had the highest signaling strength among all immune cell types in LN. A trajectory study of macrophages within LN scRNA-seq data was performed. The results indicated a macrophage transition order from macrophage sub2 to sub3 and then to sub1 along the pseudotime in LN, as shown in Fig. 2E. A heatmap showing the evolution of gene expression in macrophages over pseudotime is presented in Fig. 2F. In the heatmap, three main categories of gene sets were observed, as summarized by dendrogram clustering. The first category of genes is mainly upregulated in macrophage sub2, including WARS, S100A6, FCGR3A, and IFITM2, which are mainly inflammation-patrolling leukocyte signature genes [25]. Genes in the second category were mainly upregulated in macrophage sub3, including C1QA, CD163, and DAB2, which are phagocytic phenotype genes of macrophages [26–28]. The third category of genes mainly upregulated in macrophage sub1, including HLA-DQA1 and CD74, which are mainly related to class II major histocompatibility complex (MHC-II)-mediated antigen presentation [29]. Overall, our trajectory study predicted a transition of macrophages from inflammatory patrolling to phagocytic and then an antigen-presenting phenotype within the LN kidney.

3. Validation of macrophage distribution and their phenotypic changes in LN tissue using multiplexed immunofluorescence.

**Fig. 3:**
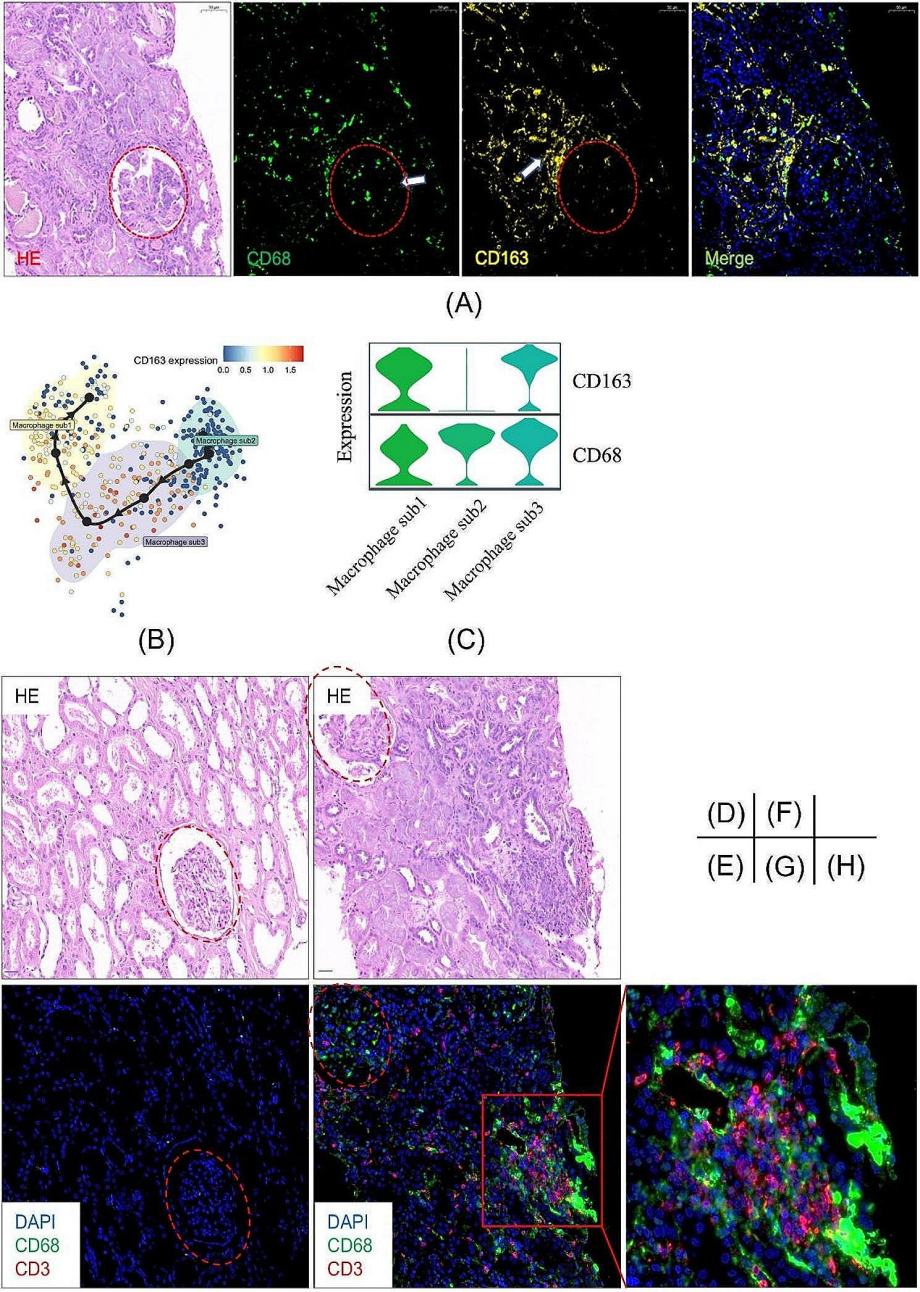
Results of the multiplexed immunofluorescence assay. (**A**) Hematoxylin and eosin (HE) staining and immunofluorescence staining targeting CD68 and CD163 in lupus nephritis (LN) tissue samples. (**B**) The gene expression of CD163 alongside the pseudotime within macrophage subtypes in LN. (**C**) The stacked violin plots of CD163 and CD68 gene expression in macrophages sub1, 2 and 3 in LN. (**D**, **E**, **F**, **G**, **H**) HE staining and immunofluorescence staining targeting CD68 and CD3 as along with DAPI staining in normal kidney tissues (**D**, **E**) and LN tissue samples (**F**, **G**, **H**). The LN immunofluorescence images in this figure are from a 39-year-old woman with 1 year of stage IV + V lupus nephritis (LN patient 5 in Supplementary Table 1)

Our multiplexed immunofluorescence results showed that CD68+ (green) macrophages were massively distributed in both the glomeruli and interstitium of LN kidneys. Macrophages within the glomeruli obviously lacked expression of CD163, whereas those in the proximal space outside the glomeruli showed high expression of CD163, as shown in Fig. 3A, which was from a 39-year-old woman with 1 year of stage IV + V lupus nephritis (LN patient 5 in Supplementary Table 1). The panoramic view of the slide was presented in Supplementary Fig. 3A and 3B. The lack of CD163 expression strongly suggested that macrophages within the glomeruli mainly belonged to the macrophage sub2 cluster, and were inflammatory patrolling macrophages stemming from the circulatory system that had just adhered and deposited in the glomeruli [25, 30]. Macrophages in the proximal space outside the glomeruli belonged to the macrophage sub3 cluster, which was characterized by high expression of CD163 and was spatiotemporally proximal to macrophage sub2. According to the results of trajectory analysis, macrophage sub1 has a significant antigen-presenting role and an intimate relationship with T cells via the MHC-II complex, as shown in Supplementary Figs. 4 and 5. Our multiplexed immunofluorescence results confirmed that clusters of CD68 + macrophages had close physical contact with CD3 + T cells within the renal parenchyma of LN, as shown in Fig. 3B. Based on our results of trajectory study of bioinformatics analysis and the multiplexed immunofluorescence assay, we summarized the evolutionary spectrum of macrophages within LN kidney tissue, as shown in Supplementary Fig. 5. Thus, we suggest that macrophages within the LN kidney play a vital role in aberrant signaling, contributing to disease development and progression.

4. The LGALS9 pathway was significantly enriched in macrophage signaling in the immune microenvironment of LN.

**Fig. 4:**
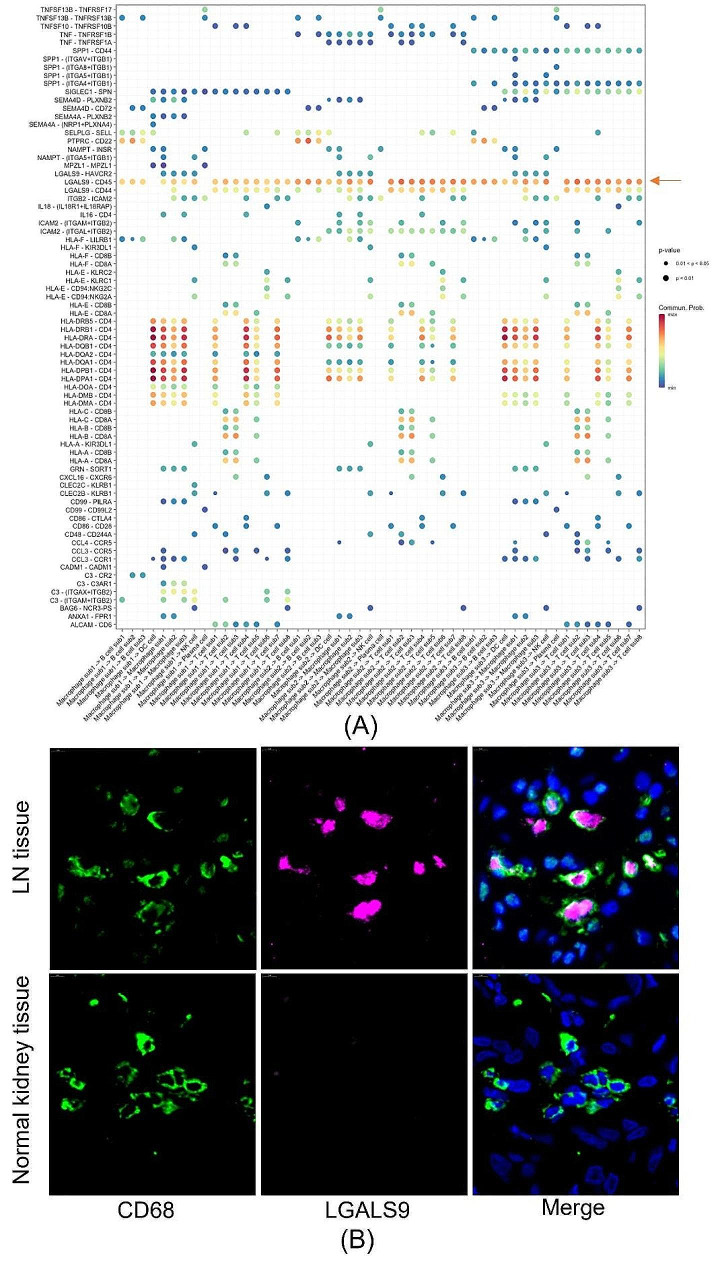
Cell‒cell interaction analysis revealed a significant Galectin-9 signaling pathway of macrophages in the immune micromilieu of lupus nephritis (LN). (**A**) Dot plot showing the signaling patterns of macrophages and other immune cells. The dot size is proportional to the contribution score computed in pattern recognition analysis. A higher contribution score implies that the signaling pathway is more enriched in the corresponding cell group. Enriched LGALS9 signaling was observed to be the most significant beside the MHC-I-CD8 and MHC-II-CD4 interactions. (**B**) The immunofluorescence study confirmed the predicted upregulation of LGALS9 in CD68 + macrophages in LN. The LN immunofluorescence images in this figure are from a 37-year-old woman with 3-year duration of LN disease, which was stage II (LN patient 3 in Supplementary Table 1)

To further elucidate the immunoregulatory role of macrophages in LN, we performed intercellular signaling pathway enrichment analysis. A dot plot of the significant signaling pairs of macrophages in LN is presented in Fig. 4A. In addition to interactions related to MHC-I and MHC-II molecules, which are classical antigen-presenting molecules, the LGALS9 signaling pathway was the most significantly enriched pathway in the immune microenvironment of LN. As presented in Fig. 4B, our multiplexed immunofluorescence results confirmed that a significant elevation of LGALS9 expression was observed in CD68 + macrophages in LN tissue samples. The panoramic view of the slide was presented in Supplementary Fig. 3C. Furthermore, our bioinformatic analysis results demonstrated a significant lupus nephritis-specific high-expression of LGALS9 in macrophages, as compared to macrophages from IgA nephropathy [9], allograft biopsy specimen [10], acute kidney injury and chronic kidney disease [11]. The results were presented in Supplementary Fig. 6 and supplementary Table 2.

After a careful literature review, we found that the role of LGALS9 in LN pathogenesis is not well documented, and its influence on macrophages is unclear. Therefore, we performed lentivirus-mediated overexpression to preliminarily evaluate the effect of LGALS9 on THP-1 cells, which is an ideal model for the functional study of monocytes and macrophages [31, 32].

5. Overexpression of LGALS9 elicited pro-inflammatory activation in THP-1 cells.

**Fig. 5:**
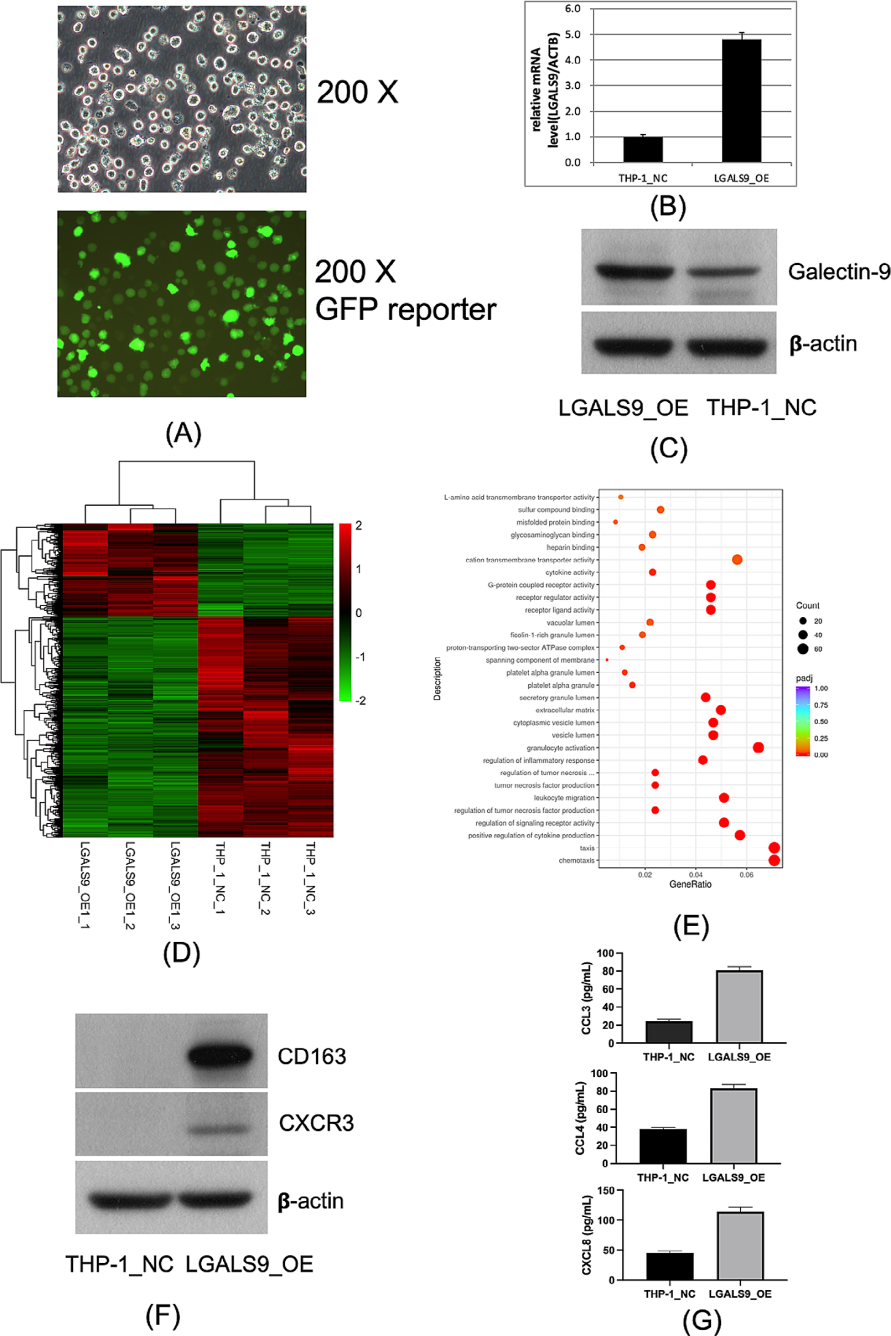
Overexpression of LGALS9 elicits a significant pro-inflammatory effect on THP-1 cells. (**A**) The fluorogram of reporter GFP confirmed a successful lentivirus packaging and infection in THP-1 cells. (**B**, **C**) LGALS9 expression was significantly elevated in mRNA and protein levels in LGALS9_OE than in control THP-1_NC cells. The bar graph represents the mean and standard deviation of the results from three experiment replicates. (**D**) Heatmap of DEGs between LGALS9_OE cells and the negative control THP_1_NC cells. (**E**) Bubble plots of the results of GO enrichment analysis of the DEGs between LGALS9_OE and THP-1_NC cells. (**F**) Western blot of CD163 and CXCR3 in LGALS9_OE and THP-1_NC cells. (**G**) ELISA results of CCL3, CCL4 and CXCL8 in LGALS9_OE and THP-1_NC. The bar graph represents the mean and standard deviation of the results from three experiment replicates cells

We overexpressed gene LGALS9 in THP-1 cells by lentiviral packaging and infection. The validation fluorogram of THP-1 cells infected with lentivirus bearing green fluorescent protein (GFP) reporter gene is shown in Fig. 5A. The LGALS9 mRNA expression in THP-1 cells overexpressing LGALS9, named LGALS9_OE cells, was found to be 4.8-fold higher of that in control THP-1_NC cells, as shown in Fig. 5B and Supplementary Fig. 7A. The expression of protein Galectin-9, which coded by LGALS9 gene, in THP-1_NC and LGALS9_OE cells was measured by western blotting and shown in Fig. 5C. The cellular transcriptome was analyzed using an RNA-seq assay. With Padj < 0.05 and |log2FoldChange| > 1 as the threshold, 449 genes were significantly up-regulated and 1063 genes were significantly down-regulated in LGALS9_OE cells. The gene expression heatmap is shown in Fig. 5D and the volcano plot is shown in Supplementary Fig. 7B. The results of GO enrichment analysis are shown in Fig. 5E, and the KEGG pathway enrichment results are shown in Supplementary Fig. 7C. The GO enrichment terms mainly included chemotaxis, positive regulation of cytokine production, leukocyte migration and regulation of inflammatory response. The KEGG pathway enrichment results mainly included phagosomes, rheumatoid arthritis, complement coagulation cascades, and antigen processing and presentation. All these pathways are well-documented to be activated in the macrophages of LN [33–35]. To further validate the RNA-seq assay results, we detected expression of classical markers of macrophage activation and pro-inflammatory chemotaxis that were significantly up-regulated in the sequencing results, such as proteins of CD163 and CXCR3, and cytokines CCL3, CCL4, and CXCL8, by western blotting and enzyme-linked immunosorbent assay (ELISA). Our results showed that overexpressing LGALS9 gene in THP-1 cells significantly elevate the CD163 and CXCR3 protein expression. The ELISA results confirmed that THP-1 cells overexpressing LGALS9 gene significantly up-regulate cytokines CCL3, CCL4 and CXCL8, which all have strong pro-inflammatory chemotactic effects on various inflammatory cells [36, 37]. Results were shown in Fig. 5F and G. Therefore, our gain-of-function assay preliminarily showed that LGALS9 can elicit a pro-LN phenotype in macrophages, and thus be a potential druggable target, which deserves further investigation.

## Discussion

Understanding the immune cells that drive inflammation in LN will advance our knowledge regarding the pathogenesis of this disease and help identify new therapeutics. An integrative analysis using both bulk transcriptome and scRNA-seq data was performed in this study. First, analysis of bulk RNA-seq data clearly showed that IFN signaling was significantly upregulated within LN and that the intensity of IFN signaling was positively correlated with macrophage infiltration in LN. Second, 17 immune cell subtypes were identified in the LN scRNA-seq data; ssGSEA quantitatively showed that macrophages contributed the most among all immune cells to the total DEGs of bulk RNA-seq data. Additionally, intercellular signal communication analysis showed that the intensity of macrophage signaling was the highest among all immune cell subtypes, suggesting that macrophages may play an important role in regulating the aberrant signaling networks in LN. Third, by integrating bioinformatics analysis and multiplexed immunofluorescence assay, this study characterized the spectrum of macrophage phenotypic evolution from inflammatory patrolling to phagocytic and, finally, to antigen-presenting phenotypes, and presented numbers of vital pro-inflammatory factors secreted by them. Fourth, the LGALS9 pathway was identified to be aberrantly enriched in macrophage-related signaling networks within LN, and that overexpressing LGALS9 in the monocyte-macrophage model THP-1 cells exerted a significant activating and pro-LN effect on the phenotype of monocytic cells; this suggested for the first time, that LGALS9 may be a potential druggable target for macrophage-targeting strategies in treating LN.

In addition to the present study, other studies have also examined the role of macrophages in LN. Renal macrophages have been found to be closely associated with LN severity and progression [38, 39]. Studies have also shown that in an acute renal injury model, pro-inflammatory CX3CR1 + macrophages are recruited to the glomeruli in a chemoattraction-dependent manner, and that TLR3 is engaged in this process. These macrophages can produce inflammatory cytokines, nitric oxide synthase, procoagulants, and matrix metalloproteinases [30, 40]. After stimulation by the IgG immune complex, these macrophages are alternatively activated, accompanied by a metabolic switch to glycolysis. Glycolytic metabolic reprogramming leads to IL-1β production in an mTOR- and HIF1α signaling pathway-dependent manner [41, 42]. These findings were in consistent with our findings that infiltrating macrophage sub2, which were mainly inflammatory patrolling macrophages located in the glomeruli, were stimulated by the autoimmune IgG complex deposited under the glomerular endothelium and transitioned to macrophage sub3 in the crescent and proximal renal tubulointerstitial region. These macrophages took on the alternatively activated macrophage phenotype marked by significant elevation of CD163 expression, along with complement and IL1β production.

Abnormal activation of the complement system is a hallmark of LN [43]. In this study, we found that macrophages had the highest local complement secretion among all infiltrating immune cells. In particular, phagocytic macrophage sub3 was the cell type with the highest C1 and C2 secretion, whereas antigen-presenting macrophage sub1 had the highest C3 secretion in the immune microenvironment of LN. A violin plot of the expression of complement system genes in LN is shown in Supplementary Fig. 8. Thus, our study presents evidence that aberrant activation of the complement system is closely associated with macrophages in LN.

Notably, we termed all CD68 + leukocytes as macrophages in the cluster annotation of scRNA-seq data in this study. Nevertheless, we found that they were the same or similar cell populations for the macrophages termed in this study and monocytes or mononuclear phagocytes in other reports [25, 44], as evidenced by their surface marker expression. In fact, all these cells belong to the mononuclear phagocyte system. The minor differences among these cells were primarily in their ontogeny and secondarily in their location [45]. Although the distinction and classification of these cell types has been challenging because of their overlapping characteristics, this is not the focus of the present study.

Our study had certain limitations. First, although we predicted a transition of macrophages from an inflammatory patrolling to a phagocytic and then an antigen-presenting phenotype within the LN kidney via bioinformatic trajectory study, which is further evidenced by our multiplexed immunofluorescence results, our data only showed that they co-existed in the kidney of LN patients. How macrophages are plastic, with three phenotypes being interchangeable, depends on the nephritis stages, therapeutic drugs, remission or relapse of systemic lupus, and many other factors. Further spatiotemporal series studies are needed to confirm how macrophages migrate and evolve in the LN kidney. Second, this study mainly focused on the role of macrophages in the immune microenvironment of LN without further analysis of other immune cells such as T cells, B cells, and NK cells; however, researchers still consider that these immune cells together with macrophages have very important roles in the aberrant immune microenvironmental network of LN. Third, although this study used the THP-1 cell model to preliminarily investigate the effect of LGALS9 on monocyte-macrophages, and its function still needs further validation at the in vivo level in LN animal models, and its molecular mechanism needs further study.

## Conclusion

The present study provided evidence of the important role of macrophages in the immune microenvironment of LN. Through intercellular signaling communication analysis and experiments using the THP-1 cellular model, this study indicated for the first time that LGALS9 has a pro-inflammatory activating effect on macrophages and that it may be a potential candidate for macrophage-targeting strategies in treating LN.

### Electronic supplementary material

Below is the link to the electronic supplementary material.

Supplementary Material 1

## Data Availability

The datasets GSE32591 and GSE183279 were sourced from the GEO database (https://www.ncbi.nlm.nih.gov/geo). The single-cell RNA sequencing (scRNA-seq) data for lupus nephritis, IgA nephropathy, and allograft biopsy specimens were obtained from the Human Cell Atlas database (https://explore.data.humancellatlas.org/projects). The RNA sequencing data for THP-1 cells overexpressing LGALS9 or a negative control is available from the authors upon reasonable request.
