# Supplementary material for: Integrative analysis of single-cell and bulk transcriptome data reveal the significant role of macrophages in lupus nephritis
Source: Arthritis Res Ther. 2024 Apr 12;26:84. doi: 10.1186/s13075-024-03311-y (PMC11010324; doi:10.1186/s13075-024-03311-y)
Supplement: Supplementary file 1 — Supplementary Material 1 [file 13075_2024_3311_MOESM1_ESM.pptx]

## Slide 1
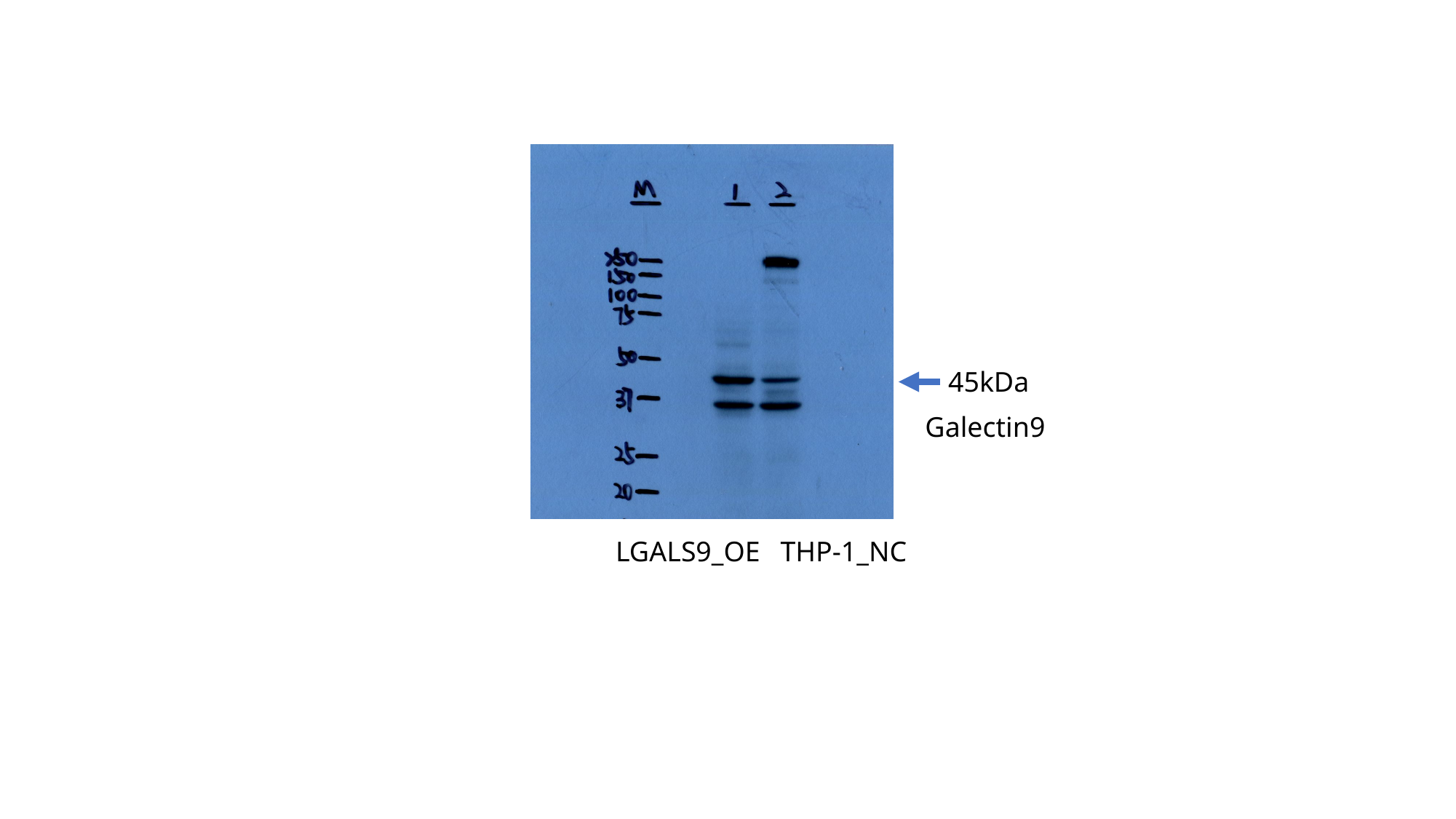

45kDa
Galectin9
LGALS9_OE
THP-1_NC

## Slide 2
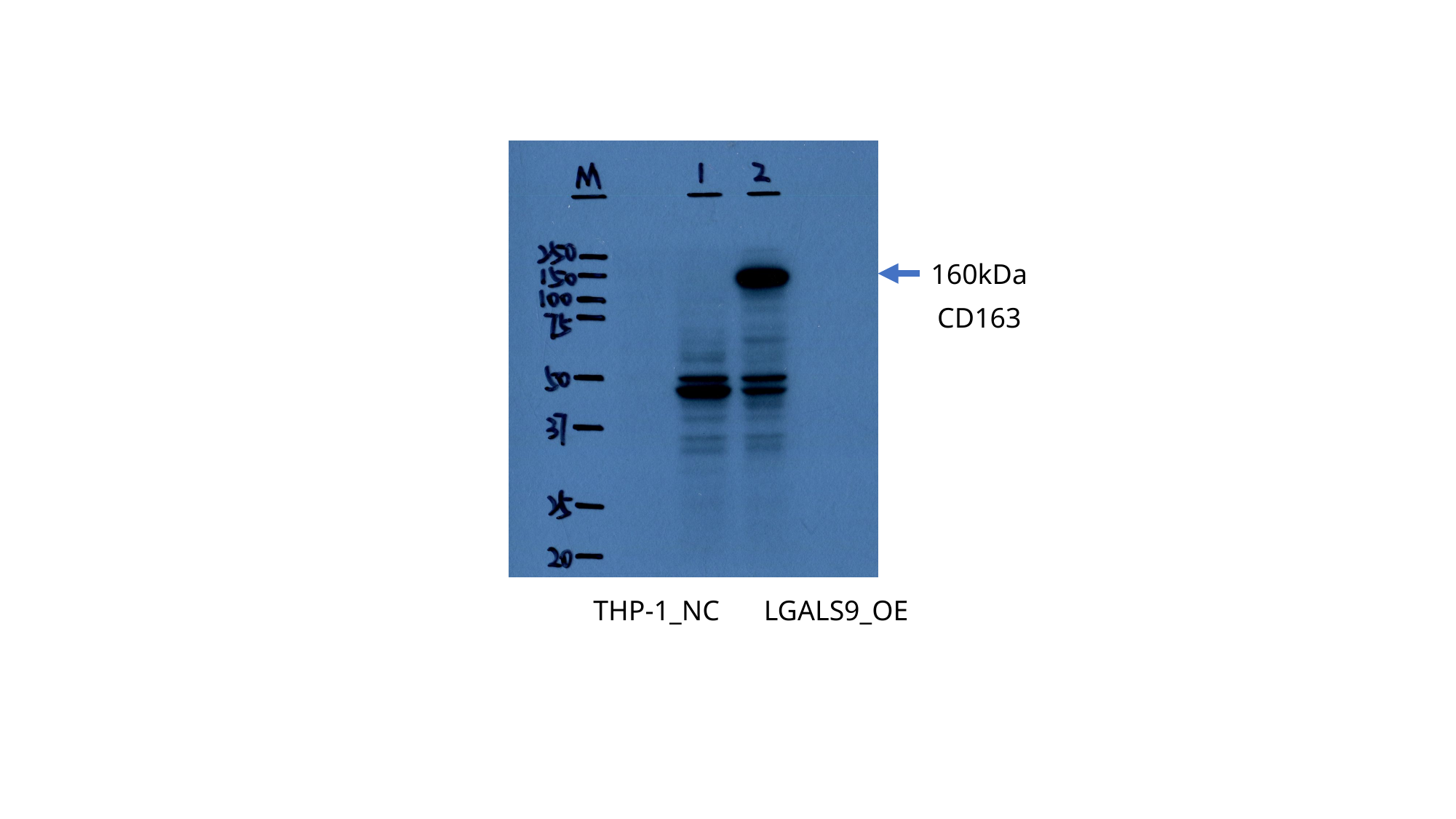

160kDa
CD163
THP-1_NC
LGALS9_OE

## Slide 3
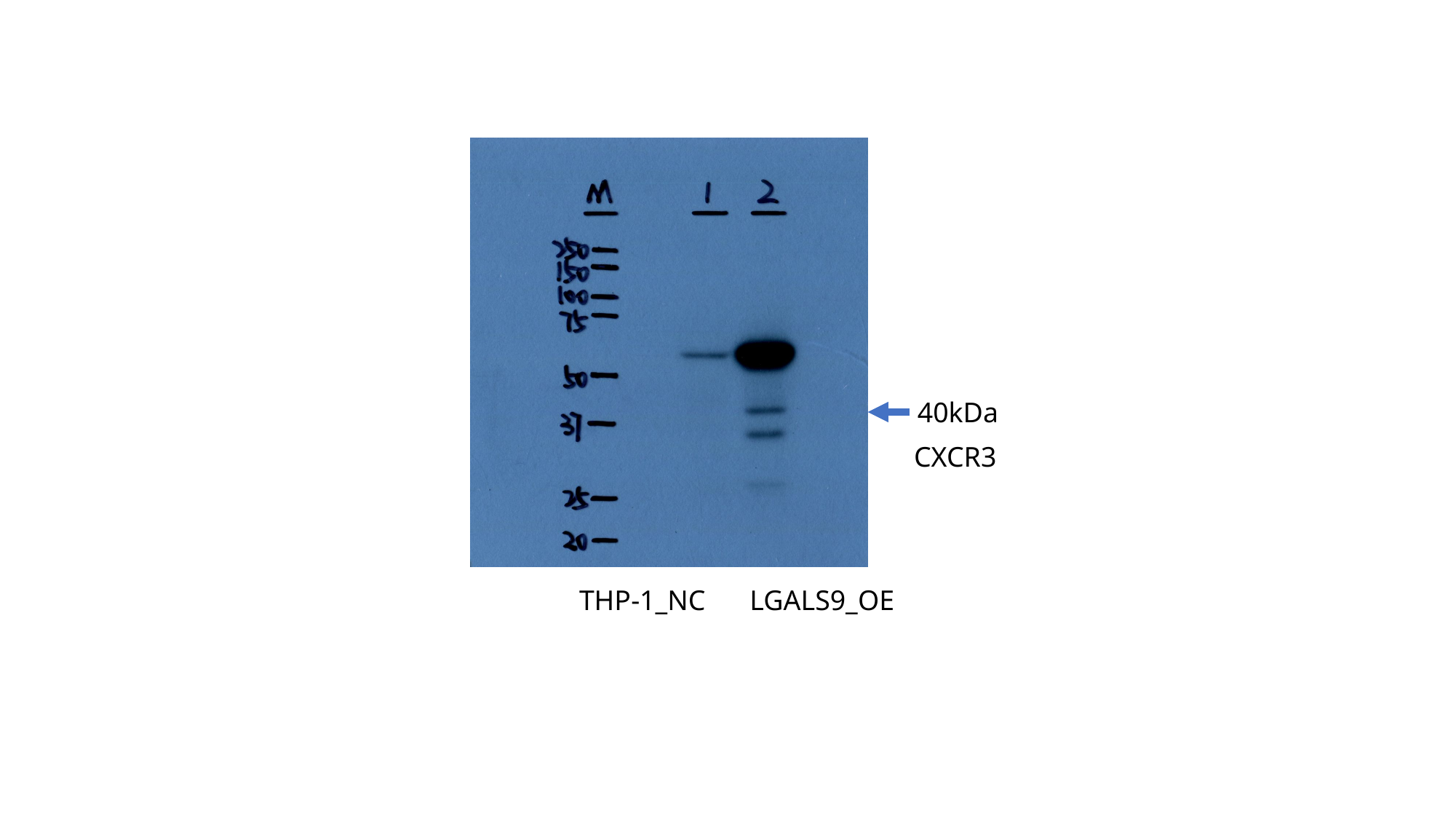

40kDa
CXCR3
THP-1_NC
LGALS9_OE

## Slide 4
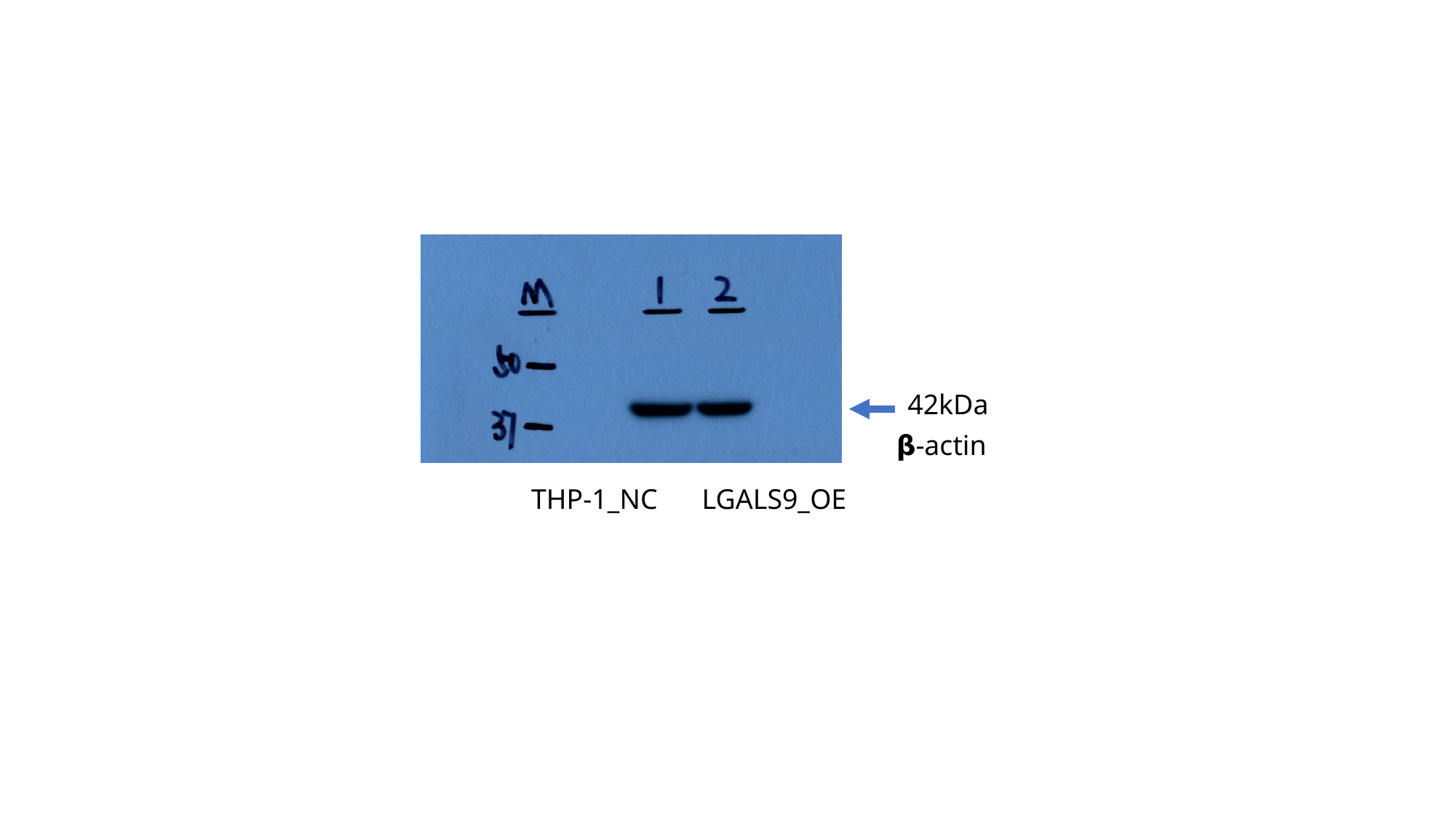

42kDa
𝝱-actin
THP-1_NC
LGALS9_OE

## Slide 5
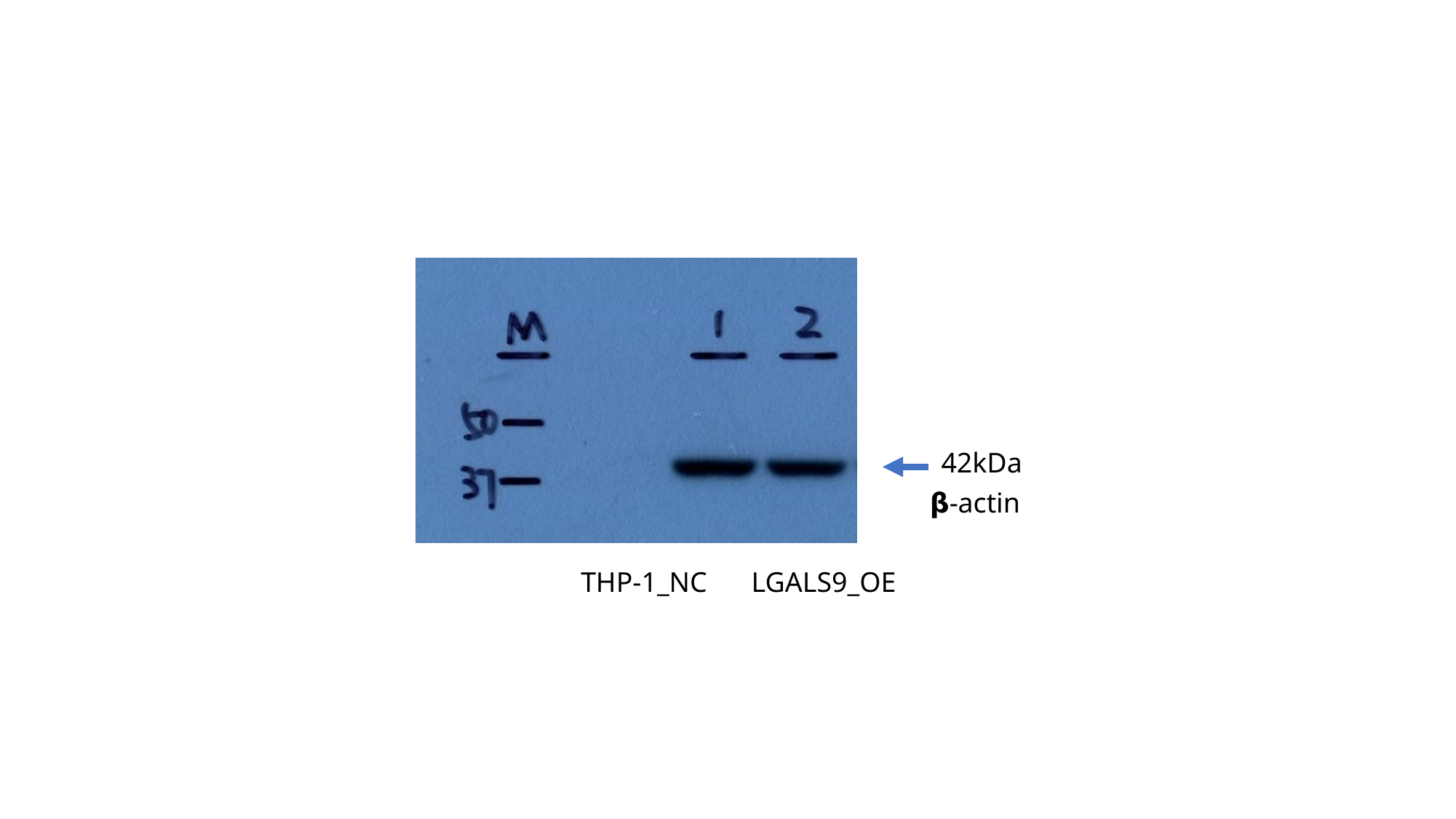

42kDa
𝝱-actin
THP-1_NC
LGALS9_OE
